# Research on the multi-physics field coupling simulation of aero-rotor blade electrochemical machining

**DOI:** 10.1038/s41598-021-92066-6

**Published:** 2021-06-17

**Authors:** Liang Huang, Yan Cao, Xinyun Zhang, Jiahao Zhang, Yan Lei, Yao Li, Qingming Fan

**Affiliations:** 1 Mechatronic Engineering, Xi’an Technological University, No.2 Xuefu Middle Street, Weiyang District, Xi’an, 710021 China; 2 Tool Structure Manufacturing, Xi’an KunLun Industry (Group) Company With Limited Liability, No.67 Xingfu North Street, Xincheng District, Xi’an, 710021 China

**Keywords:** Electrochemistry, Mechanical engineering

## Abstract

Aimed at the problem that the quality of the formation of aero-rotor blades with complex shaped structures is difficult to predict due to the coupling of each physical field in the process of electrochemical machining, a mathematical model of electrochemical machining characterized by the conductivity of the multi-physics field is established firstly by studying the coupling action mechanism of the flow field and temperature field on electric field conductivity in the machining process. Then, utilizing the Runge–Kutta method, the relationship between the conductivity and the machine path is analysed; Finally, based on this relationship, the multi-physics field electrochemical machining erosion model is compared with the traditional single electric field electrochemical machining erosion model. The results show that the error of the multi-physical field coupling prediction model proposed in this study is in the range of 1.27–2.35%, while the accuracy of the single electric field prediction model is in the range of 4.35–5.88%. The theoretical value of the multi-physics field coupling simulation is closer to the measured value of the test and can accurately simulate the actual electrochemical machining process, which can provide a theoretical basis for the design of cathode tools and parameter optimization in the actual processing process and is of great significance to improve the quality and efficiency of the electrochemical machining of aero-rotor blades.

## Introduction

As the main heart of aero-rotor engines, aero-rotor blades are listed as the first key part due to their harsh environment of high temperature and complex stress for a long time. The performance level of aero-rotor blades, especially temperature-bearing capacity and mechanical strength, has become an important symbol of the engine advanced degree^1^. With the development of aero-rotor engines in recent years towards the direction of high thrust-weight ratio structures and new lightweight super-alloy materials^2^, a higher requirement for surface machining technology of aero-rotor blades has been proposed. In terms of subtractive manufacturing: on the one hand, due to the high cost of the existing high-speed NC milling (numerical control machining) technology in the research and development of special cutting tools and wear compensation for complex thin-walled structure products with high-temperature cemented carbide material characteristics, this point-contact cutting method can easily cause the deformation of the surface of thin-walled parts by producing high local contact stress, which ultimately affects the forming profile and structural properties of blades^3^. On the other hand, although EDM (electric discharge machining) can avoid the inconvenience caused by contact milling by non-contact discharge corrosion, the recast layer and thermal stress/thermal deformation caused by arc high-temperature discharge on the machined surface also cause the rotor blade to be easily affected by the corresponding behaviours through material modification^4^. However, in additive manufacturing technology, the complexity of the design and manufacture of the whole disk casting die and the high cost characteristics brought by the use of the SLM/SLS (selective laser melting/Selective Laser Sintering) technology also make it difficult to achieve a higher breakthrough in the high precision and low cost manufacturing of the rotor blade^5,6^.

In electrochemical machining technology, as a kind of non-contact subtractive manufacturing, the free ions of the workpiece anode and the tool cathode surface under the external power supply are oxidized and reduced in the electrolyte, respectively. This leads the material on the anode surface of the workpiece to be dissolved in the electrolyte in the ion form of the lost electron, and the electrolyte ion on the tool cathode surface causes the electron to produce hydrogen, to remove the corrosion of the anode workpiece. With the advantage of no loss of tool cathodes and the design method of reverse copy cathode, this technology can produce arbitrarily complex shape structures with new lightweight super-alloy materials at low cost and efficiently. However, due to the complexity and variability of the electrochemical etching process, different process parameters directly affect the mass transfer process of the electrochemical reaction in the inter-electrode gap through the designed flow channel structure, and the electrochemical mass transfer process in the inter-electrode gap also determines the temperature field distribution of the electrochemical reaction in each part. The temperature field and flow field distribution in each part in the inter-electrode gap determine the corresponding electric field distribution; On the other hand, different process parameters will change the physical field by changing the partial field source parameters of each physical field in the above process, and the final macroscopic erosion process is determined by the multi-physics field coupling model which integrates the above two factors. To establish the mapping relationship of this key part by a mathematical theory model to form accurate guidance on this aspect, some scholars have performed the following research: S.Van Damme and J.Deconinck of the University of Brussels, proposed a time-dependent numerical model of the electric-flow field, which combined with the electric neutral condition and the approximate linear temperature relationship under the polarization of the electrode–electrolyte interface to simulate the effect of electrolyte mass transfer (migration, diffusion and convection) on current density^7–9^; Based on this, Professor F.Klocke of RWTH Aachen, realized the establishment of an interdisciplinary simulation coupling model for electrochemical machining by using the conservation equations of electric field, fluid flow and heat transfer, and combining the analytical approximate function of the influence of temperature and gas evolution on conductivity, and the reliability of the model was verified by the construction rules of the inverse modelling method^10^; Meanwhile, Dr. Chen Yuanlong of Hefei University of Technology established a coupling model based on the flow field and temperature field through the basic physical field model established by Deconinck, and realized the electrolytic processing process analysis of a fusion multi-physical field^11^; Based on the above research, it was found that there are some problems in the establishment and analysis of the multi-physics field coupling model: that is, most of the previous studies in the multi-physics field focus on only the qualitative characterization of erosion quality by different process parameters, but there is no clear quantitative relationship between the different process parameters and each physical field source parameter, the coupling of multi-physical fields and the final corresponding macroscopic erosion quality. To solve the above problems, this study analysed the interaction among the electric field, flow field and temperature field during electrochemical machining, and the change in conductivity was taken as the bridge between the interaction guidance of each physical field. The coupling model of multi-physics field electrochemical initial machining characterized by the change in conductivity is established, and the distribution law of each field source parameter along the processing path is analysed by the Runge–Kutta method. Then, based on the initial distribution law of each physical field, using mobile grid physical field (ALE-Arbitrary Lagrange–Euler method) in COMSOL Multi-physics software, the finite element analysis of the electrolytic blade under multi-physical field coupling is carried out. Finally, results show that the value of the proposed multi-physics field coupling simulation method is closer to the actual processing value, and the proposed model can accurately reflect the actual electrochemical machining process, which is of great significance to improve the electrochemical machining quality and efficiency of aero-rotor blades.

## Multi-physics field coupling model for electrochemical machining of aero-rotor blades

### Analysis of the characteristics of each physics field in electrochemical machining

**Figure 1:**
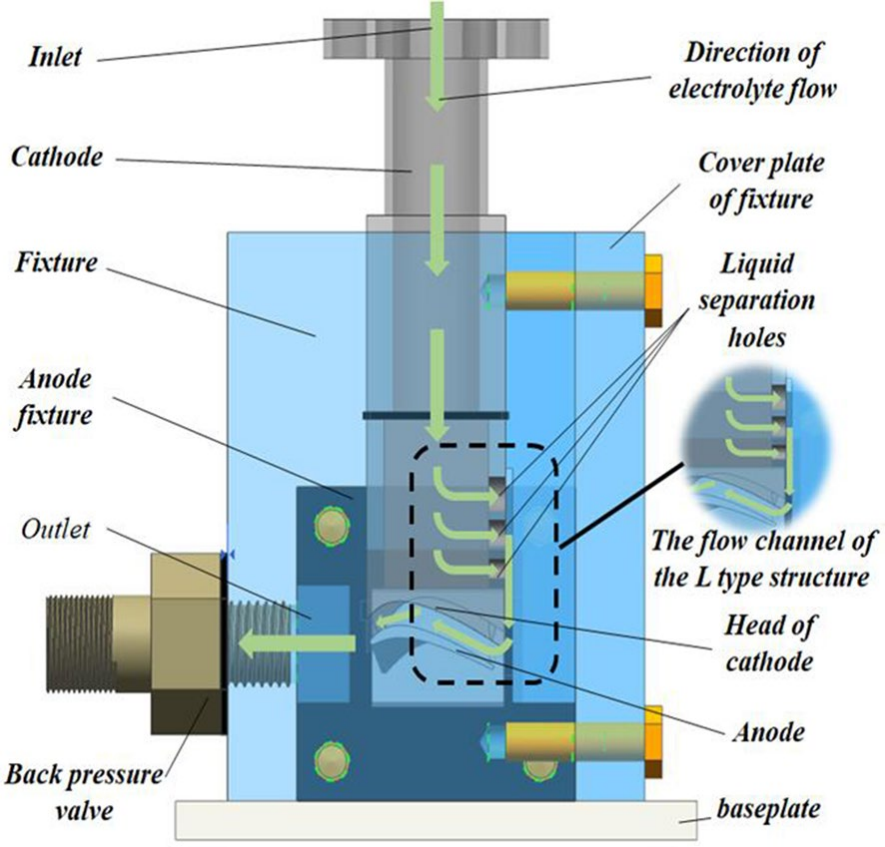
Schematic diagram of assembly of the electrolytic machining fixtures for aero-rotor blades.

In this study, the blade of a small aero-rotor engine is taken as the research object. Considering that both the positive flow processing the mode of electrolyte and the reflux flow processing mode of the electrolyte are needed to design the corresponding liquid guide holes on the cathode tool, and the formation of this hole will interfere with the uniformity and consistency of the flow field in the adjacent cathode surface, which will easily lead to turbulence and hole problems in this part and reduce the overall forming quality of the blade surface, the side flow processing mode of the electrolyte can avoid the above problems because it can ensure the stability of electrolyte distribution in the cathode surface^12^. Furthermore, considering that the surface of the aero-rotor blades is composed of NURBS curves (Non uniform rational B-spline) in different spatial positions superimposed along the axial direction, the whole blade surface presents a complex spatial surface structure with large twist variation along the axial direction. Therefore, in the side flow processing mode of the electrolyte, if the electrolyte flow direction is parallel to the axial direction of the blade, the electrolyte is easily isolated or passively shunted by the marginal plateau, and the curvature of the electrolyte flows through the surface under the longer flow along the path is large, which will cause the whole flow field to appear disordered, unstable and controllable, so it can not satisfy the high precision processing of the blade. However, if the electrolyte flow direction is perpendicular to the axial direction of the blade, because the blade width is much smaller than the length of the blade axis, and the curvature of each electrolyte flows through the blade surface is small, on the one hand, the flow field distribution is more uniform and easier to control, but on the other hand, the electrolytic product and heat can be discharged quickly. Therefore, this study chooses the side flow processing mode of electrolyte flow perpendicular to the blade axis (the corresponding whole assembly design structure is shown in Fig. 1), and combined with the finite element method, the following physics field is established.

#### Flow field

**Figure 2:**
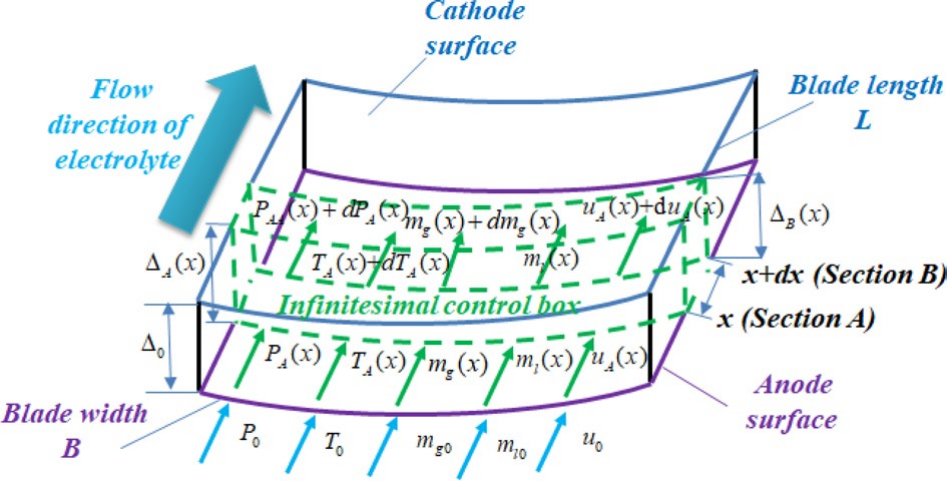
Construction of the flow field model for aero-rotor blades by the infinitesima method.

Due to the low content of bubbles produced by electrochemical reactions in the initial stage of electrolytic machining and the small effect on the liquid phase in the inter-electrode gap^11^, according to the mass conservation and continuity law of gas–liquid two-phase flow formed in the inter-electrode gap, the mass conservation continuity model of the gas phase and liquid phase was established by using the infinitesimal method (as shown in Fig. 2). Formula (1) indicates that the mass of the liquid phase on any section is equal to the mass of the liquid phase on the inlet of the inter-electrode gap. Furthermore, as shown in Fig. 2, when the electrolyte flows from the inlet and flows out from the liquid separation holes and enters the inter-electrode gap, the initial flow rate of the electrolyte changes in this L flow channel due to the local head loss effect. Therefore, the flow rate of the electrolyte into the initial inter-electrode gap is calculated and recorded as *u*_0_ by combining the continuous equation and the local head loss equation of electrolyte flow.

Liquid phase:

1 $$\rho _{l} u_{0} \Delta _{0} B = \rho _{l} u_{A} (x)(1 - \beta _{A} (x))\Delta _{A} (x)B = \rho _{l} u_{B} (x + dx)(1 - \beta _{B} (x + dx))\Delta _{B} (x + dx)B$$

where, $\rho _{l}$ represents the electrolyte density (g/cm^3^); $u_{0}$ represents the flow velocity of the electrolyte when the electrolyte enters the inter-electrode gap (cm/s); $\Delta _{0}$ represents the inter-electrode gap when the electrolyte enters the inter-electrode gap (cm); $u_{A} (x)$ represents the flow velocity of the electrolyte when the electrolyte passes through section A (cm/s); $\Delta _{A} (x)$ represents the inter-electrode gap when the electrolyte passes through section A (cm); and $\beta _{A}$ represents the void fraction when the electrolyte passes through the section A (%).

Formula (2) indicates that the cathode precipitates gas and accumulates along path *x* (cm), which increases the gas mass passing along each section and increases linearly with *x*, the higher the current density *i* is, the higher the gas increase rate.

2 $${\text{Gas phase}}:\frac{{P_{A} (x)M_{g} }}{{R_{g} T_{A} (x)}}\beta _{A} (x)u_{A} (x) = \eta _{g} K_{g} ix$$

where, $P_{A} (x)$ represents the gas pressure when the electrolyte passes through section A (Mpa); $R_{g}$ represents the universal gas constant (J/(mol K)); $M_{g}$ represents the gas molar mass (g/mol); $T_{A} (x)$ represents the gas temperature when the electrolyte passes through section A (K); $\eta _{g}$ represents the current efficiency of precipitated gases (%); $K_{g}$ represents the mass electrochemical equivalent of gas (g/C); and $i$ represents the input current density (A/cm^2^).

In terms of momentum conservation, in addition to the work done by the pressure potential energy of the fluid, because the electrolyte resists the relative motion among the adjacent flow layers through viscous force in the inter-electrode gap, and through the mechanical conduction among the adjacent layers, the velocity distribution in the actual flow layers is different from that of the ideal fluid. The velocity near the solid–liquid boundary decreases gradually, the velocity away from the solid–liquid boundary increases gradually, and this viscous force is mainly represented by viscous shear force. In addition, considering that the mass of the liquid is much larger than that of the gas, the sum of the mass of the liquid and the mass of the gas is replaced by the mass of the liquid (that is,$m_{l} + m_{g} \approx m_{l}$). Based on the Viscous Bernoulli’s equation, the momentum conservation model of the corresponding flow field is established as follows:

3 $$\rho _{l} \Delta _{0} u_{0} \frac{{du_{A} (x)}}{{dx}}{\text{ + }}xP_{A} (x)\frac{{d\Delta _{A} (x)}}{{dx}}{\text{ + }}x\Delta _{A} (x)\frac{{dP_{A} (x)}}{{dx}} = {\text{ - }}P(x)\Delta (x) - 2\tau {\text{(}}x)x - \eta _{g} K_{g} iu_{A} (x)\Delta _{A} (x)$$

$$\tau (x) = 0.0332\rho _{l} \left[\frac{{(1 - \beta (x))^{3} u(x)^{7} v}}{{\Delta (x)}}\right]^{{0.25}}$$

where, $\tau$ represents the shear stress (Mpa); $\mu$ represents the dynamic viscosity of flow (Mpa·s), that is $\mu = \rho v$; and $v$ represents the kinematic viscosity (Mpa·s).

As the electrolyte flows in the inter-electrode gap, the fluid will consume some energy to overcome the above motion resistance under the action of the viscosity and shear force in the flow layer, and this part of the energy will be converted into heat loss. Combined with Darcy's equation, an expression for this part of the frictional head loss ($H_{f}$) is as follows:

4 $$H_{f} = \lambda \frac{L}{\Delta }\frac{{u^{2} }}{{2g}}m_{l} g$$

where, $\lambda$ represents the friction loss factors of the fluid; $L$ represents the path of the electrolyte along the inter-electrode gap (cm); and *g* represents the acceleration of gravity (m/s^2^).

According to formulas (3)–(4), by introducing the potential energy and kinetic energy of the fluid in the electrochemical machining process and correlating with the Joule heat and enthalpy in the temperature field, the energy conservation model of aero-rotor blade electrochemical machining is established as follows:

5 $$T_{A} (x) - T_{0} = \frac{1}{{m_{l} C_{l} }}\left(U_{R} iB\Delta _{A} - \lambda m_{l} \frac{{u_{A}^{2} (x) - u_{0}^{2} }}{{2\Delta _{0} }}\right)x - 0.5m_{l} \frac{{u_{A}^{2} (x) - u_{0}^{2} }}{{\Delta _{0} }}$$

where, $C_{l}$ represents the specific heat capacity of electrolyte fluid (J/g K), $u_{A} (x)$ represents the flow velocity of the electrolyte when the electrolyte passes through section A (cm/s); *x* represents the processing path (cm); *i* represents the input current density (A/cm^2^), and $U_{R}$ represents the voltage between the electrodes (V).

#### Temperature field

In the process of electrochemical machining, the electrolyte flowing in the inter-electrode gap is equivalent to the resistance. When the two poles are electrified, the Joule heat generated by the current flowing through the electrolyte resistance is the heat source in the inter-electrode gap, thus forming the temperature field, and the corresponding Joule heat ($P_{h}$(J)) calculations are as follows:

6 $$dP_{h} = U_{R} iB\Delta _{A} dx$$

#### Electric field

Because the two electrode surfaces of the rotor blades are Gaussian curved surfaces, the electric potential distribution between the two electrodes satisfies the Laplace equation. Furthermore, the law of anode erosion in the inter-electrode gap satisfies Faraday's law, so the mathematical model of the electric field is obtained as follows:

7 $$\begin{aligned} & \nabla ^{2} \phi {\text{ = }}0 \\ & \frac{{\partial ^{2} \phi }}{{\partial x^{2} }} + \frac{{\partial ^{2} \phi }}{{\partial y^{2} }} + \frac{{\partial ^{2} \phi }}{{\partial z^{2} }} = 0 \\ & \Delta _{n} = \frac{{\eta \omega \kappa (U{\text{ - }}U_{P} {\text{ - }}U_{c} )}}{{v_{c} }} = \frac{{\eta \omega \kappa U_{R} }}{{v_{j} \cos \theta }}(i = \kappa (x)U_{R} /\Delta (x), v_{c} = \eta \omega i) \\ \end{aligned}$$

where, $v_{c}$ represents the corrosion speed (cm/s);$v_{j}$ represents the feed speed (cm/s); $\theta$ represents the angle between the cathode feed rate and the surface normal vector of a point on the anode surface (°); $U$ represents the input voltage (V); $U_{P}$ represents the voltage from electrochemical polarization (V);$\kappa$ represents the electrolyte conductivity (S/cm); and $U_{c}$ represents the electrolyte conductivity (V).

### Analysis of the characteristics of the multi-physics field in electrochemical machining

**Figure 3:**
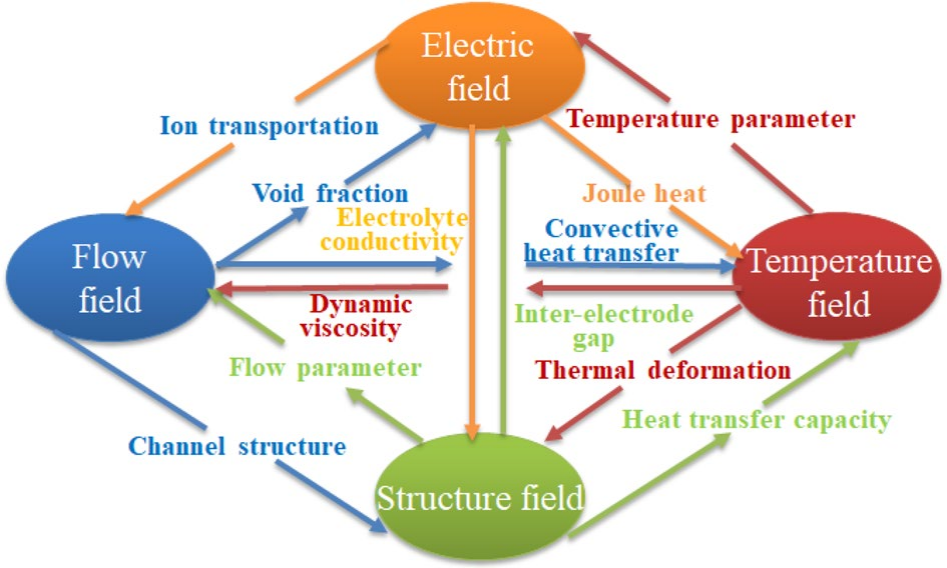
Multi-physics field mechanism.

By bringing the electric field model and the temperature field model into each conservation model of the flow field, it was found that the temperature field is mainly changed by the field source parameters of the flow field through energy conservation, while the void fraction of the flow field and the temperature of the temperature field affect the final erosion quality through the electrolyte conductivity (as shown in Fig. 3). Moreover, combined with the influence of temperature and void fraction on conductivity in reference^9^, the mathematical model (as shown in formula (9)) of electrochemical machining characterized by the conductivity of the multi-physics field is established by introducing formula (8) into formulas (1)–(7). In addition, based on the DIASINKER vertical electrochemical machining machine tool provided by Xi’an KunLun Industry (Group) Company (the corresponding equipment parameters are shown in Table 1), the equivalent resistance partial voltage of electrochemical polarization and concentration difference polarization were calculated to be 1.15 V and 0.78 V by electrochemical polarization test and rotating disk electrode test, respectively. Then, by studying the electrochemical reaction of IN 718 alloy in NaNO_3_ electrolyte, the current efficiency of hydrogen precipitation is converted from the ratio of actual corrosion to theoretical corrosion of IN 718 alloy, and the corresponding current density and input voltage range are obtained based on this; Finally, using the temperature rise model of electrolyte flow and Reynolds number criterion, and the turbulent flow of electrolyte in the inter-electrode gap is realized under the premise of preventing the electrolyte from boiling when the temperature is too high, and based on Bernoulli's equation, a feasible range of field source parameters is established. Then, the optimal initial parameters for the multi-physics field solution in this study are determined by orthogonal testing and using the numerical simulation technique to ensure the uniformity of the physical field distribution (including the flow velocity, flow and pressure distribution of the flow field and the electrolyte current density of the electric field), so the initial process parameters in the inter-electrode gap are obtained as shown in Table 2.

8 $$\kappa \left( x \right) = \kappa _{0} \left[ {1 + \xi \left( {T(x) - T_{0} } \right)} \right] \cdot \left( {1 - \beta (x)} \right)^{n} {\text{ }}$$

9 $$\left\{ {\begin{array}{*{20}l} {u_{0} \Delta _{0} = u\left( x \right)\left[ {1 - \beta \left( x \right)} \right]\Delta \left( x \right)} \\ {\frac{{P(x)M_{g} }}{{R_{g} T(x)}}\beta (x)u(x) = \eta _{g} K_{g} ix} \\ {\rho _{l} \Delta _{0} u_{0} \frac{{du(x)}}{{dx}}{\text{ + }}xP(x)\frac{{d\Delta (x)}}{{dx}}{\text{ + }}x\Delta (x)\frac{{dP(x)}}{{dx}} = {\text{ - }}P(x)\Delta (x) - 2\tau (x)x - \eta _{g} K_{g} iu(x)\Delta (x)} \\ {T(x) - T_{0} = \frac{1}{{m_{l} C_{l} }}(U_{R} iB\Delta (x) - \lambda m_{l} \frac{{u^{2} (x) - u_{0}^{2} }}{{2\Delta _{0} }})x - 0.5m_{l} \frac{{u^{2} (x) - u_{0}^{2} }}{{\Delta _{0} }}} \\ {\kappa \left( x \right) = \kappa _{0} \left[ {1 + \xi \left( {T(x) - T_{0} } \right)} \right] \cdot \left( {1 - \beta (x)} \right)^{n} } \\ {\tau (x) = 0.0332\rho _{l} [\frac{{(1 - \beta (x))^{3} u(x)^{7} v}}{{\Delta (x)}}]^{{0.25}} } \\ \end{array} } \right.$$

**Table 1:** ECM experimental equipment parameters.

Plant parameters	Values
Anode material	IN 718 alloy
Cathode material	Red copper
Volume electrochemical equivalent (IN 718) ($\omega$)	123.79 mm^3^/(A h)
Initial inter-electrode gap ($\Delta _{0}$)	0.3 mm
Feed speed (*v*_*j*_)	0.3 mm/min
Composition of electrolyte	20% NaNO_3_

**Table 2:** ECM experimental process parameters.

Plant parameters	Values
Voltage from electrochemical polarization (*U*_P_)	1.15 V
Voltage from concentration polarization (*U*_c_)	0.78 V
Current efficiency of precipitated hydrogen ($\eta _{g}$)	65%
Initial flow velocity ($u_{0}$)	18 m/s
Initial inlet pressure ($P_{0}$)	0.8 Mpa
Molar mass of hydrogen ($M_{g}$)	2.014 g/mol
Universal gas constant ($R_{g}$)	8.3144 J/mol K
Mass electrochemical equivalent of hydrogen ($K_{g}$)	2.371 [g/(A h)]
Electrolyte density ($\rho _{l}$)	1157 kg/m^3^
Kinematic viscosity of electrolyte ($v$)	0.8 × 10^−6^ m^2^/s
Initial conductivity of electrolyte ($\kappa _{0}$)	12.5 S/m
Initial processing temperature/℃ ($T_{0}$)	25 $\pm$ 2
Friction loss factors of electrolyte ($\lambda$)	0.045
Initial void fraction/% ($\beta _{0}$)	0
Specific heat capacity of electrolyte ($C_{l}$)	4125 (J/kg K)
Input voltage (*U*)	15 V
Current density (*i*)	40 A/cm^2^
Current efficiency ($\eta$)	65%

**Figure 4:**
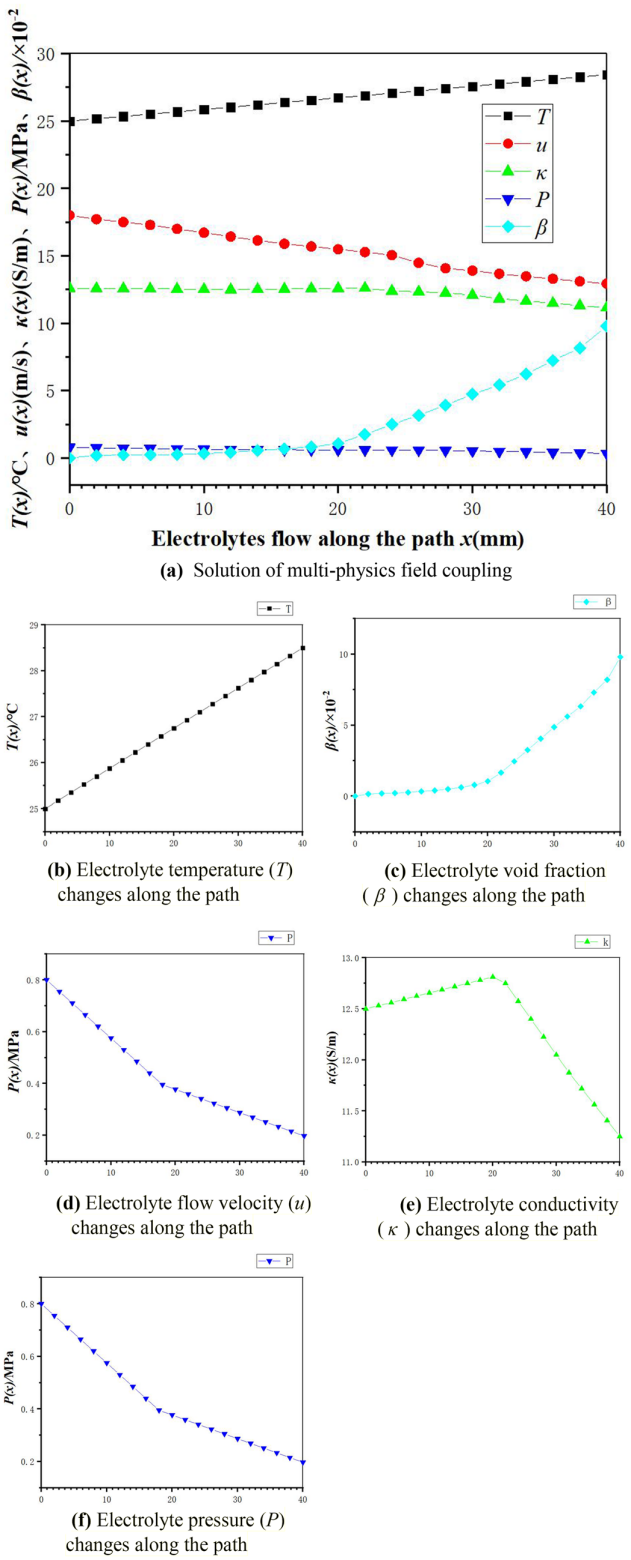
(**a**) Solution of multi-physics field coupling. (**b**) Electrolyte temperature (*T*) changes along the path. (**c**) Electrolyte void fraction ($\beta$) changes along the path. (**d**) Electrolyte flow velocity (*u*) changes along the path. (**e**) Electrolyte conductivity ($\kappa$) changes along the path. (**f**) Electrolyte pressure (*P*) changes along the path.

According to the variation in the field source parameters along the path in Fig. 4a (the corresponding temperature unit is converted by K to degree Celsius, and the length unit is converted by cm to mm), in order to explain the changes of the field source parameters, the Origin software fits the field source parameters evolution with the path to obtain the corresponding subpicture (as shown in Fig. 4b–f).

For temperature (*T*) and void fraction ($\beta$), with the continuous progress of inter-electrode electrochemical machining, the thermal energy and bubble generated by the electrochemical reaction of the electrode is transmitted to the outlet port with the liquid phase mass transfer, and formed accumulation. The numerical fitting found that the *T* increases linearly along the path (as shown in Fig. 4b), while the bubbles form a nonlinear increase trend due to the accumulation at the outlet port (as shown in Fig. 4c). When the electrolyte flows into the inter-electrode gap, considering that there is no bubble at the inlet port, and combined with the viscosity shear stress calculation formula in formula (8), it was found that the flow velocity (*u*) mainly affects the viscosity shear stress ($\tau$) at this time, and under the high flow velocity, the *u* gradually decreases with the path (*x*) and the corresponding flow velocity reduction is higher (as shown in Fig. 4d); However, with the gradual increase of the $\beta$ along the path, the increase of the $\beta$ that subdominates the viscosity shear force will hinder the increase of the viscosity shear force, and the $\tau$ decreases together with the *u*, and finally shows the gradual decrease of the flow velocity reduction. Meanwhile, in the diagram of the change of electrolyte conductivity ($\kappa$) with the path (as shown in Fig. 4e), and combined with the electrolyte conductivity calculation formula (8), it was found that the $\kappa$ at the inlet port increases slowly mainly determined by the *T* due to the almost absence of bubbles; With the continuous increase of the $\beta$, the corresponding $\kappa$ decreases significantly with the dominant $\beta$ together with the accumulation *T*. In addition, in the diagram of the change of electrolyte pressure (*P*) with the path (as shown in Fig. 4f), due to the high flow velocity in the inlet port, and combined with the Bernoulli conservation principle, the corresponding *P* is relatively low, meanwhile, combined with the above energy conservation formula (9) and kinetic energy conservation formula (9), it was found that due to the high decline degree of *u*, the corresponding kinetic energy increment gradually increases, which making the corresponding pressure potential energy gradually increases, and finally shows the decrease of *P* along the path; while at the outlet port, because the change of *u* is small, the corresponding kinetic energy increment is small and eventually the reduction of the *P* gradually decreases.

Moreover, the coupling effect of these field source parameters on the change in each physics field is finally characterized by a mass erosion model in an electric field by the change in electrolyte conductivity ($\kappa$) (as shown in Fig. 3). Therefore, according to the process parameters and field source parameters obtained in this part, the initial processing structure can be optimized and improved, to verify the feasibility and accuracy of the model through the following physics field simulation analysis.

## Multi-physics field coupling simulation of aero-rotor blade electrochemical machining

### Construction of a multi-physics field coupling simulation analysis strategy

**Figure 5:**
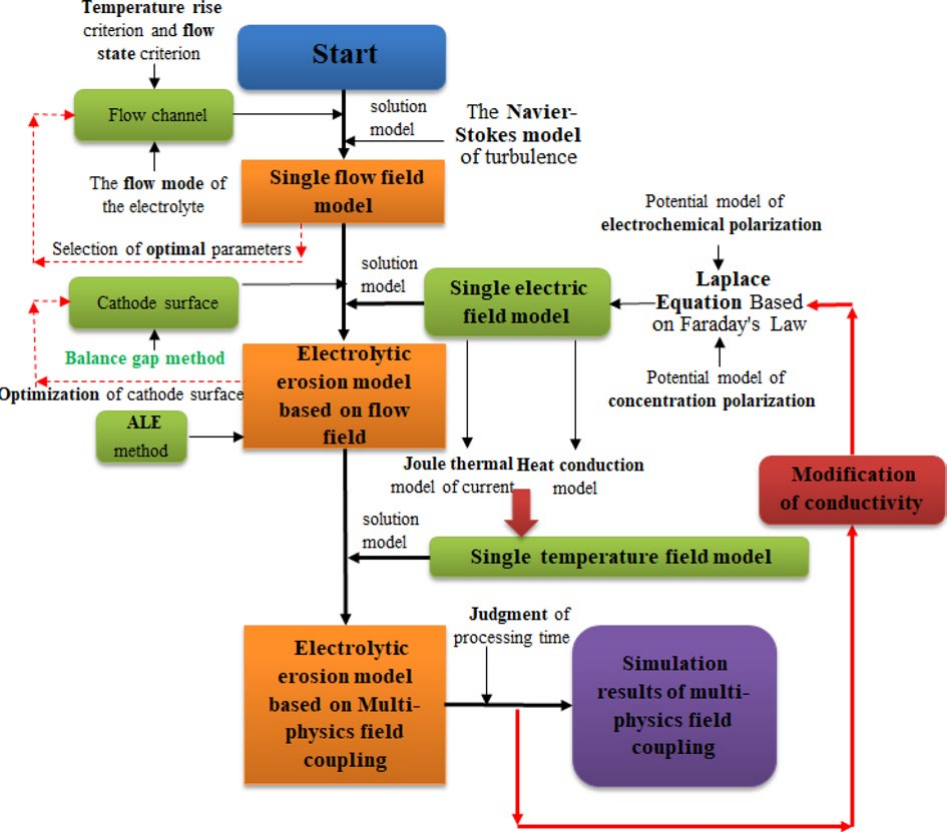
Steps of the multi-physics field coupling simulation.

Considering that there is no highly nonlinear interaction among the physical fields in the process of electrochemical machining, the sequential coupling method is used to analyse the single physical field in this study, that is, the single physical field is solved first, and then the multi-physical field coupling simulation is completed in an orderly manner by using the coupling variable transfer relation combined with the physical field relations in Fig. 3. Meanwhile, in COMSOL software, the time-dependent solver provides three different time-stepping methods: the implicit BDF (backward differentiation formula) and Generalized alpha methods and the explicit Runge–Kutta methods. On the one hand, the main idea of BDF method is based on the linear multi-step method, approximating the function derivative through a given function value of the given time, thus improving the accuracy of the approximation. However, the severe damping effects produced at the lower order lead to its main applicability for solutions of order 1 to 3. On the other hand, the Generalized alpha method is similar to the BDF method, but the underlying technique is different. The method contains a parameter (called α in the literature) to control the degree of high-frequency damping. Compared to the backward differential formula, the Generalized alpha method has smaller damping and hence more accurate, while the stability is also poor for the same solution method reasons. Considering the Runge–Kutta method as a class of explicit iterative algorithms for solving nonlinear differential equations, it uses Lagrange interpolation ideas to construct higher order iterative calculation formulas through continuous interpolation to obtain high precision numerical solutions of nonlinear differential equations. Therefore, combined with the fine machining of the aero-blades, this paper using the higher order (4 orders 5 times) Runge–Kutta method to perform the solution. In addition, the purpose of the simulation of the aero-rotor blade electrochemical machining process is to predict the final forming profile of the anode workpiece, so the final foothold of the simulation is to solve the actual electric field distribution in the processing zone. It is necessary to determine the conductivity distribution of the electrolyte in the processing area, which is related to the variation in the flow field parameters and temperature field parameters along the path in Fig. 4. Therefore, on the basis of the direct action of a steady flow field on an electric field, the relationship of the temperature field can be introduced to realize the accurate simulation of the final erosion state (the simulation flow is shown in Fig. 5).

According to the results of the multi-physics field coupling calculation shown in Fig. 4, the field source parameters of each physical field are related to the path. This will lead to different changes in conductivity with the path in different positions of aero-rotor blade electrochemical machining. Thus, the erosion of each part of the blade is inconsistent. Considering that the Lagrange method (which describes the motion boundary with high accuracy, but reduces the element solving accuracy easily by generating mesh entanglement when dealing with large mesh deformation) and the Euler method (which has high element solving accuracy when dealing with large mesh deformation, but can not accurately solve the motion boundary) have their own problems in describing the finite element mesh motion, this study used the arbitrary Lagrangian–Euler method (ALE). By introducing a reference configuration that can be independent of the real configuration and the initial configuration, the ALE method is degraded into the Lagrange method and Euler method to address blade grid motion during the blade surface erosion process as follows:

1. When the computational grid is fixed in space, the Euler method is used;
2. When the computational grid motion with matter, the Lagrange method is used;
3. When the computational grid free motion, the ALE method is used;

Furthermore, to prevent grid discontinuity, the function of the automatic redivision mesh is added and the normal uniform feed of the cathode boundary *v*_n_ is set, thus matching the dissolution velocity equation:

10 $$v_{n} = \frac{{dx}}{{dt}}n$$

Electrochemical machining is actually the process of gradual dissolution of the anode, so the differential equations of continuous electrochemical machining can be expressed as follows:

11 $$dl = v_{n} (t)dt$$

where, $dl$ represents the increment of dissolved displacement in *dt* time; $v_{n} (t)$ represents the normal dissolution rate of the anode erosion surface; and *dt* represents the time increment.

Then the whole electrochemical machining processes is discretized with time as the variable, that is, Δ*t* is taken as the step size and *t*_*p*_ (*p* = 1,2,…*n*) is taken as a time series, and the displacement of the anode solution surface is obtained as follows:

12 $$\Delta l_{p} = v_{n} (t_{p} )\Delta t$$

Therefore, the position change relationships of the anode solution surface are as follows:

13 $$\left\{ {\begin{array}{*{20}c} {x_{{a_{{p + 1}} }} = x_{{a_{p} }} + \Delta l_{p} \cos \gamma _{a} \cos \alpha } \\ {y_{{a_{{p + 1}} }} = y_{{a_{p} }} + \Delta l_{p} \cos \gamma _{a} \sin \alpha } \\ {z_{{a_{{p + 1}} }} = z_{{a_{p} }} + \Delta l_{p} \sin \gamma _{a} } \\ \end{array} } \right.$$

In summary, the ALE method can be used to accurately simulate the instantaneous erosion of various parts of the blade under the action of different conductivities in the process of electrochemical machining.

### Analysis of multi-physics field coupling simulation results

**Figure 6:**
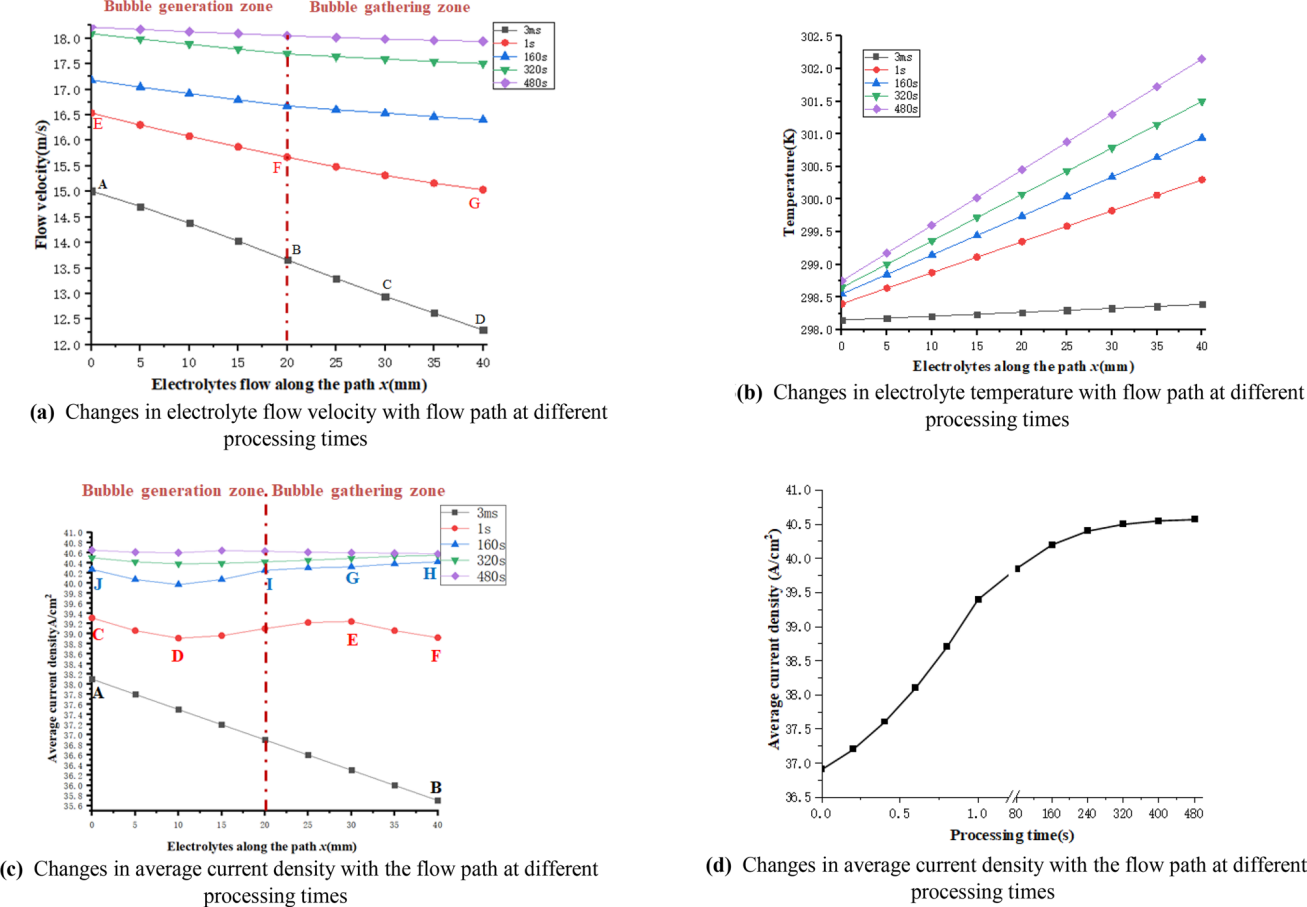
(**a**) Changes in electrolyte flow velocity with flow path at different processing times. (**b**) Changes in electrolyte temperature with flow path at different processing times. (**c**) Changes in average current density with the flow path at different processing times. (**d**) Changes in average current density with the flow path at different processing times.

This study takes the finish machining of concave concrete as an example, and sets the processing time to 480 s, so the simulation process is carried out by using the above method, and the following results are obtained (as shown in Fig. 6):

According to Fig. 6a, when the first electrolyte flows into the machining gap (3 ms), since the bubble rate at the whole path is very small, the electrolyte flow rate mainly determines the viscosity shear force compared with that of the bubble rate. Therefore, it is observed that the velocity change from AB to BC to CD is gradually decreases (under the gradual decrease of the viscosity shear force). As the processing time progresses, because the bubbles form an accumulation from the outlet direction to the inlet direction, at this time, the bubble rate of the electrolyte and the flow velocity jointly determine the viscosity shear force, and the effect on the electrolyte flow rate is gradually reduced (as shown by EF and FG in Fig. 6a). When the processing time is more than 160 s, the change of bubble rate on electrolyte flow velocity is gradually significant, and the flow velocity of each part tends to be balanced at 480 s. According to Fig. 6b, the electrolyte temperature increases linearly along the flow path (this further verifies the correctness of the model solution in Fig. 4), and the slope of the linear change in temperature increases with the increase in processing time, indicating that with the continuous processing, the heat produced by the electrochemical reaction is gradually transferred with the flow path through the mass transfer in liquid phase mode, which makes the inlet and outlet have a large temperature difference, and forming an uneven change in temperature along the path. It can also be observed that the slope of temperature growth decreases with the continuous advance of processing time, which also indicates that the processing tends to balance gradually and that the relevant parameters of each physical field remain constant. As shown in Fig. 6c, when the first electrolyte flows into the machining gap (3 ms), the average current density on the anode surface decreases gradually with the flow path, and the average current density decreases to the same degree at each position (as shown in AB in Fig. 6c). The main reason is that void fraction and temperature have little effect on electrolyte conductivity and the main factor of conductivity is flow velocity. It can also be seen that with processing, the middle section curve gradually protrudes (as shown by DE in Fig. 6c), indicating that the current density in the middle section is higher than that in both sides. The main reason for this phenomenon is that the concave has curvature characteristics that changes with the flow path which results in excessive electrolyte accumulation in the middle part of the concave surface and higher current density (the conclusion of the back blade is opposite), so the erosion rate of different parts of the workpiece surface is different. The main reason for the decrease in the EF section is that the effect of the bubble rate on conductivity is dominant compared with that of temperature, and the EF section has less electrolyte accumulation than the DE section. As a result, the current density decreases. In subsequent processing, the effect of temperature on the conductivity at the outlet is still increasing, which makes the EF section with lower conductivity at the outlet increase after 160 s (as shown by GH in Fig. 6c). Subsequently, the corresponding bubble rate gradually moves the accumulation from the bubble gathering zone to the bubble generation zone (that is, the EF section transfers to the GI section), so that the electrolyte conductivity of the GI section increases slowly along the path under the common influence of the electrolyte temperature gradually increasing and the bubble rate gradually accumulation; while the corresponding IJ section has a higher conductivity due to the mainly higher electrolyte accumulation temperature compared to the CD section. In the subsequent machining, with the continuous accumulation of bubble rate and temperature along the path and under the combination action of its interaction, the conductivity of each path gradually consistent and reached the machining balance.

**Figure 7:**
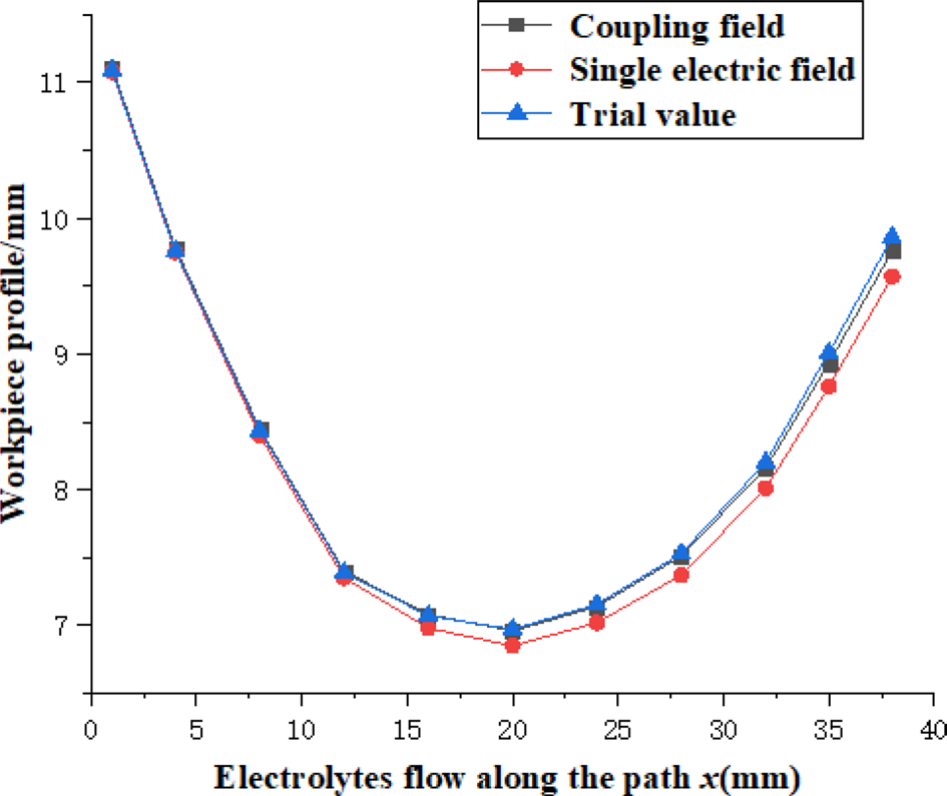
Comparison of simulation results and trial values (local).

Because the electrochemical machining stage is divided into the initial unbalanced processing stage and the balanced processing stage, to accurately predict the blade profile, this study provides statistics on the time when the sampling points of the above concave surface reach machining equilibrium. The equilibrium state diagram of concave electrochemical machining is drawn as shown in Fig. 6d. The average current density of the blade changes greatly in the initial stage of processing, while the current density changes more smoothly in the later stage of processing, and reaches the machining balance in 480 s after processing, where the average current density is 40.4 A/cm^2^. Thus, the machining is divided into 480 s of nonequilibrium stage and 480 s of the processing equilibrium stage (that is, the parameters of each physical field are constant) for joint simulation, and the simulation results of the multi-physical field and single electric field are compared (as shown in Fig. 7). According to Fig. 7, the error of the two profiles is small at the inlet, and the error along the flow path becomes larger. It can be inferred from the analysis of the blade structure and processing process that the main cause of the error is that the influence of the temperature field and flow field on the electric field conductivity under the special structure of the blade is ignored, and the single electric field simulation does not consider the influence of the bubble rate and temperature on the electrochemical machining process. Therefore, the single electric field simulation has a larger conductivity and larger anodic erosion than the coupling field simulation, and there is a large error at the outlet of the concave surface.

### Verification test of aero-rotor blade electrochemical machining

**Figure 8:**
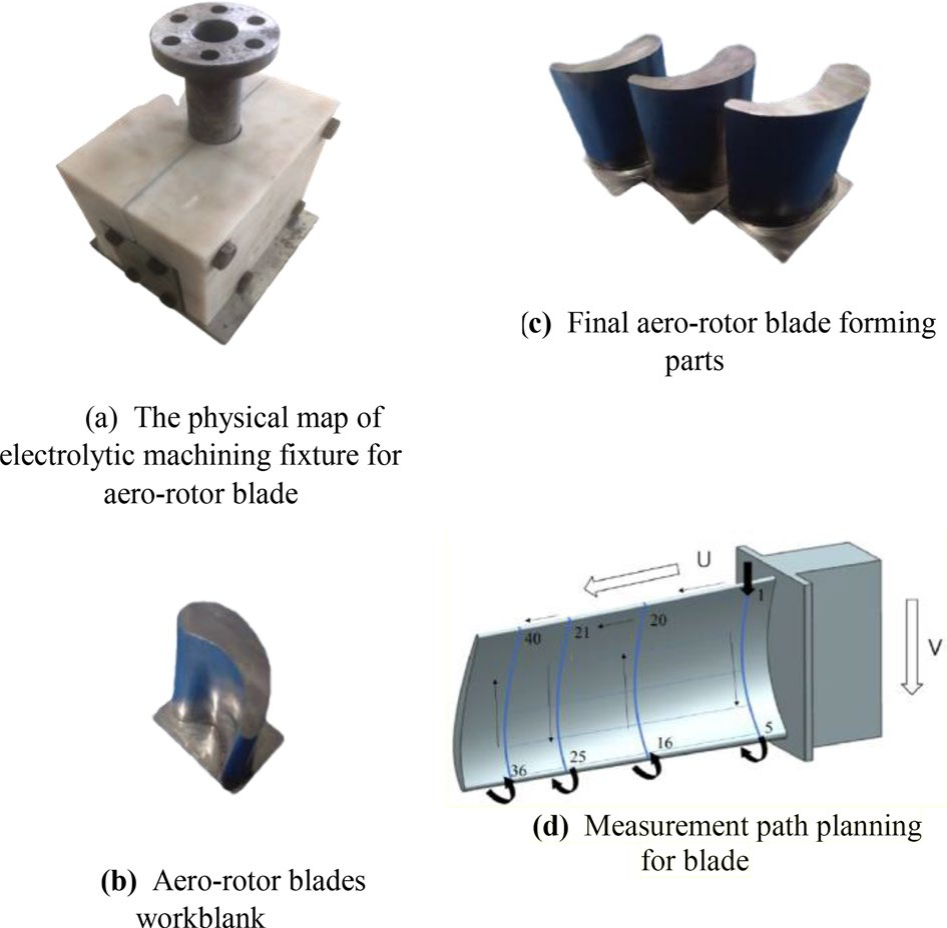
(**a**) The physical map of electrolytic machining fixture for aero-rotor blade. (**b**) Aero-rotor blades workblank. (**c**) Final aero-rotor blade forming parts. (**d**) Measurement path planning for blade.

**Figure 9:**
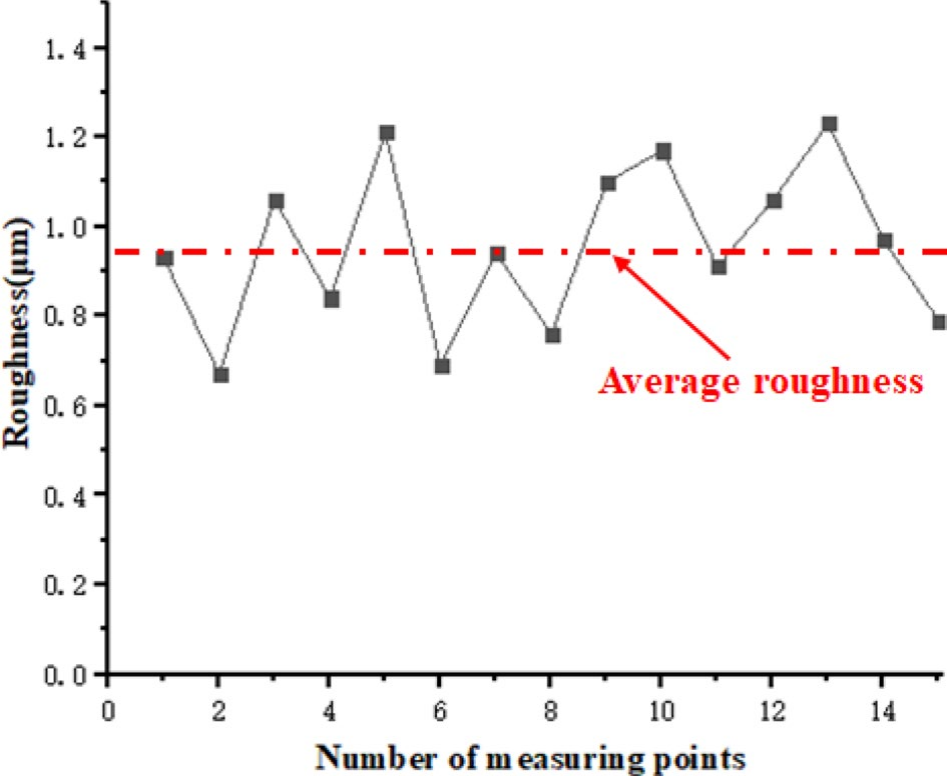
Surface roughness of the final aero-rotor blade forming parts (local).

To further verify the accuracy of the multi-physical field coupling model proposed in this study, the electrolytic machining fixture for aero-rotor blades of Fig. 1 (the physical map is shown in Fig. 8a) and the experimental parameters of Tables 1 and 2 are used, and the aero-rotor blade near-formed parts (as shown in Fig. 8b) are processed and the final blade forming parts are obtained as shown in Fig. 8c. In this paper, to ensure the rationality of the sampling, a comprehensive sampling method with equal distance along the blade body direction and equal arc length along the blade profile direction is adopted to carry out sampling path planning as shown in Fig. 8d (taking 4 control lines in the direction of the blade body U, and each control line take 5 points along the equal arc length in the V direction in the blade basin surface, then take 5 points along the V equal arc length on the back of the blade side, and enter the next control line after above testing, so take 40 points for the whole blade surface). Finally, using the ultra-high precision coordinate measuring machine (model: Leitz Reference Xi), the path planning and measurement of the sampling points of the above blades are carried out, and combination with formula (14) the surface profile prediction error diagram (as shown in Fig. 7) is drawn. Meanwhile, formula (15) can be used to calculate the prediction error for each sampling point. In in addition, the corresponding surface roughness measurement (model: ICQ 150) results are shown in Fig. 9.

14 $$d = \left[ {(x_{m} - x_{n} )^{2} + (y_{m} - y_{n} )^{2} + (z_{m} - z_{n} )^{2} } \right]^{{1/2}}$$

where, $x_{m} ,y_{m} ,z_{m}$ represents the predicted point coordinates of simulation results; $x_{n} ,y_{n} ,z_{n}$ represents the point coordinates of actual formed parts.

15 $$\varepsilon = \frac{{\left[ {\left| {\frac{{(x_{m} - x_{n} )}}{{x_{n} }}} \right| + \left| {\frac{{(y_{m} - y_{n} )}}{{y_{n} }}} \right| + \left| {\frac{{(z_{m} - z_{n} )}}{{z_{n} }}} \right|} \right]}}{3} \times 100\%$$

where, $\varepsilon$ represents the error rate between each simulation prediction point and the actual measurement point.

By comparing the model prediction value with the actual value in Fig. 7, it is found that the theoretical value of the single electric field simulation, the theoretical value of the simulation after multi-physical field coupling and the contour of the data measured by the machining test tend to be basically the same. With the increase in the electrolyte flow path, the error gradually becomes larger, and the theoretical value of the multi-physical field coupling simulation is closer to the actual measured value, mainly because the conductivity is interpreted to be constant in single electric field simulation, and the effect of gap temperature and void fraction on conductivity is not considered. At the back of the flow path, the corrosion value is smaller than the simulation value, which is caused by the repeated use of the electrolyte from the machining gap to the recovery through the machine tool base during the actual processing process. This makes the ion concentration of the electrolyte gradually decrease. At the same time, considering that the ion concentration diffusion of the electrolyte decreases gradually with the increase in the flow path, this leads to the decrease in the current density at the outlet position compared with other parts, so this part is not modified in the simulation model, which leads to such a difference in results. Finally, comparing the sampling points of the concave surface and the back of the blade with the test points of the corresponding final formed parts, the error of the multi-physical field coupling prediction model proposed in this study is in the range of 1.27–2.35%, while the accuracy of the single electric field prediction model is in the range of 4.35–5.88%, so the multi-physical field prediction model it has more stable and accurate simulation ability. Since above model has not considered the role of the ion concentration on the multi-physics field conductivity as mass transfer in liquid phase decreases along the path, the model is more suitable for the erosion mass prediction along the short processing path.

## Conclusion

Based on COMSOL Multi-physics software, the multi-physical field coupling simulation analysis is carried out to predict the profile of the blade electrochemical machining parts accurately, and the following conclusions are obtained:

1. According to the numerical function image, as the mass transfer in liquid phase continues, the bubble rate and temperature form the accumulation from the outlet to the inlet, and through the coupling effect of the bubble rate and the flow velocity on the viscous shear force, the flow velocity distribution characteristics along the whole path are ultimately determined. Meanwhile, the bubble rate of the flow field and the temperature of the temperature field are the main reasons that affect the conductivity of the electrolyte, and the change of the conductivity determined the final erosion.
2. In electrochemical machining, a multi-physical field coupling model is established based on considering the flow channel structure of the workpiece, and through the coupling of the flow field and the temperature field in the specific flow channel structure, the final erosion model with varying time domain is constructed and the coupling action analysis of the relevant field source parameters on the conductivity is performed..
3. The theoretical value of the simulation has some certain error with the measured value, but the theoretical value of the multi-physical field coupling simulation is closer to the actual measurement value, which can simulate the actual electrolytic processing process more accurately than the single field theoretical value. And the effectiveness of the simulation model is proved by comparing the experimental and theoretical models (the error of the multi-physical field coupling prediction model proposed in this study is in the range of 1.27–2.35%, while the accuracy of the single electric field prediction model is in the range of 4.35–5.88%). Since above model has not considered the role of the ion concentration on the multi-physics field conductivity as mass transfer in liquid phase decreases along the path, the model is more suitable for the erosion mass prediction along the short processing path.

## Data Availability

The data used to support the findings of this study are available from the corresponding author upon request.
